# Consistent Honor, Persistent Disadvantage: American Indian and Alaska Native Veteran Health in the National Survey of Veterans

**DOI:** 10.1177/08982643211014034

**Published:** 2021-06-24

**Authors:** Kimberly R. Huyser, Sofia Locklear, Connor Sheehan, Brenda L. Moore, John S. Butler

**Affiliations:** 1 Department of Sociology, 8166 University of British Columbia, Vancouver, BC, Canada; 2 Department of Sociology, 170285 University of New Mexico, Albuquerque, NM, USA; 3 School of Social and Family Dynamics, 7864 Arizona State University, Tempe, AZ, USA; 4 Department of Sociology, 12292 State University of New York at Buffalo, Buffalo, NY, USA; 5 Department of Sociology and Management, 12330 University of Texas at Austin, Austin, TX, USA

**Keywords:** American Indian and Alaska Native peoples, veterans, self-rated health, activities of daily living limitations

## Abstract

**Objective**: To examine self-rated health and activities of daily living (ADLs) limitations among American Indian and Alaska Native (AI/AN) veterans relative to white veterans. **Methods**: We use the 2010 National Survey of Veterans and limit the sample to veterans who identify as AI/AN or non-Hispanic white. We calculated descriptive statistics, confidence intervals, and used logistic regression. **Results**: AI/AN veterans are younger, have lower levels of income, and have higher levels of exposure to combat and environmental hazards compared to white veterans. We found that AI/AN veterans are significantly more likely to report fair/poor health controlling for socioeconomic status and experience an ADL controlling for age, health behaviors, socioeconomic status, and military factors. **Discussion:** The results indicate that AI/AN veterans are a disadvantaged population in terms of their health and disability compared to white veterans. AI/AN veterans may require additional support from family members and/or Veteran Affairs to address ADLs.

## Introduction

American Indian and Alaska Native peoples (henceforth AI/ANs) have participated in the US military since the American Revolution, and their substantial contributions have been noted (Department of Defense, 2010). Indeed, AI/ANs are overrepresented among US veterans, and, among post-9/-11 period veterans, AI/ANs have a higher percentage (18.6%) served compared to veterans of all other races (14.0%; Department of Veterans Affairs, 2012, 2017). However, much of our knowledge of AI/AN veteran health has been focused on mental health diagnosis and treatment and also on providing culturally competent services (Brooks et al., 2013; Kaufman et al., 2013; Koo et al., 2015; Noe et al., 2014; Scurfield, 1995; Shore & Manson, 2004; Shore et al., 2009). Less is known about how the health of AI/AN veterans is compared to other groups. Other research that has analyzed racial and ethnic disparities in the health of veterans has often excluded AI/AN peoples (Sheehan et al., 2015), with one recent exception that focused on rural and urban AI/AN veteran health disparities (Kaufman et al., 2020). We extend the literature on Native veteran health through examining the self-rated health and functional disability—measured by activities of daily living (ADLs) limitations—relative to non-Hispanic white male veterans (henceforth white).

### AI/AN Participation in US Military

The military is a large federal provider of employment, occupational training, and educational opportunities; post-service benefits, military experience, socialization, and training are expected to generally positively impact civilian socioeconomic attainment (Sykes & Bailey, 2020). With significant variations by service era and by race and ethnicity, veterans have been shown to have a socioeconomic status advantage over nonveterans (Sampson & Laub, 1996; Teachman & Call, 1996; Teachman & Tedrow, 2004, 2007; Teachman & Tedrow, 2004, 2007). Extant research suggests that for the general population, regardless of demographic background, motivation to join the military usually fall within four major reasons: family tradition, financial benefits, identifying with a warrior mentality, and an escape (Hall, 2011; Wertsch, 1991).

American Indian and Alaska Native veterans serve disproportionately in the US military. About 12,000 AI/ANs served in the US military during World War I, and in World War II, more than 44,000 AI/ANs served in the military (Holiday et al., 2006). Much like their numbers, their contributions were unique: in both World War I and World War II, AI/AN soldiers who spoke their tribal language were used by the US military to communicate secretly between military units (Meadows, 2009). Navajo Code Talkers created an unbreakable military code for communication of military plans, directly contributing to success of the US military forces (Meadows, 2009). More than 42,000 AI/ANs served in the Armed Forces in the Vietnam War (Holiday et al., 2006). Currently, nearly 2% of the present 1.4 million active duty military are AI/ANs, with overrepresentation among Navy and Marines (Holiday et al., 2006). Notably, AI/ANs comprise only 1.5% (AI/ANs, alone or in combination) of the US population (Norris et al., 2012).

The literature suggests that the major motivations for AI/ANs to enlist in the military seem to be pursuit of economic or educational benefits, allegiance to the United States, and family, tribal, or cultural values and traditions. Indeed, extant research ranging from studies on World War II to Desert Storm suggests that AI/ANs join the military because of the prospect for economic or educational advancement after service (Holm, 1996; Ledesma, 2007; Morningstorm, 2004). A qualitative study of Native veterans in the Southwest found that participants viewed military service itself as an educational process, and many used their military experience and veteran benefits to complete college degrees (Tsinnajinnie, 2013). Yet, like other racial minorities who served in World War II, AI/AN veterans did not have the same access to the Veterans Affairs (VA) benefits, like home loans, compared to their white counterparts (Keeler, 2016). The US Department of Defense found that Native veterans have lower levels of income, were less likely to own a home, and more likely to be unemployed than other veterans (Holiday et al., 2006). American Indian and Alaska Native men and women veterans have some of the highest rates of screening positive for housing instability through the Veterans Health Administration homeless programs (Montgomery et al., 2020). American Indian and Alaska Native veterans also have 1.9 times higher odds of being uninsured compared to their white counterparts (Johnson et al., 2010).

Tribal and cultural values have also been cited as a potential reason for AI/AN participation in the armed forces and are articulated as a desire to participate in the warrior tradition of one’s tribe or nation, which, as noted earlier, is a similar motivation as other participating groups (Ledesma, 2007; Morningstorm, 2004). The modern military provides a mechanism for AI/ANs to adapt and recreate a new warrior tradition based on traditional warrior values (Holm, 1996). Particularly, AI/AN veterans of the US military may use their experiences to revive Indigenous military societies, which provide traditional sociocultural ways of honoring and recognizing veterans (Meadows, 1999). For example, after World War II, certain Plains tribes with strong warrior traditions were revived by AI/AN veterans of the US military (see Holm, 1985).

### AI/AN Health Status

In general, AI/AN peoples have strikingly worse health outcomes than whites. AI/ANs have high rates of chronic illness and have a mortality disadvantage at every age compared to whites (Lillie-Blanton & Roubideaux, 2005; Kunitz et al., 2014). American Indian and Alaska Native peoples also seem to bear a high burden of functional disability (Moss et al., 2004). In a national study of functional limitations, 28% of AI/ANs over the age of 45 reported an ADL limitation (Fuller-Thomson & Minkler, 2005). The National Survey on Disability has found that 32% of AI/AN respondents reported some sort of limitation, highest of any group in the survey, and even 15% of AI/ANs in the age range of 18–44 years indicated some limitations in physical functioning (Altman & Rasch, 2003). This suggests that AI/ANs experience disability younger than other populations. Regarding self-rated health, AI/AN men are more likely to report fair/poor health compared to white men (Denny et al., 2005).

Unfortunately, in addition to experiencing a health disadvantage compared to whites, AI/ANs often have lower socioeconomic status than whites, which negatively influences health outcomes (Huyser et al., 2010; Link & Phelan, 1995). American Indian and Alaska Native peoples tend to have lower levels of education and income, have lower returns on education (Gregory et al., 1997; Sandefur & Liebler, 1997; Snipp, 1986; 1989; Snipp & Sandefur, 1988), and have higher rates of poverty relative to comparable whites (Huyser et al., 2014). Furthermore, AI/AN households that live near tribal lands are more likely to have lower educational attainment, higher poverty rates, a greater prevalence of female-headed households, and higher fertility rates than AI/AN households that reside in metropolitan areas with no tribal lands (Liebler, 2004; 2010; Sandefur & Liebler, 1997; Snipp, 1989).

Similar to the literature from the general population, AI/AN male veterans seem to carry a higher burden of disease and have lower socioeconomic status relative to white male veterans. Among male veterans, AI/AN veterans tend to have higher rates of mental health disparities than whites (Koo et al., 2015). Approximately 37.5% of AI/AN veterans have a disability, including service-related disability, compared to 29.1% of all other veterans (Department of Veterans Affairs, 2012, 2017). However, it is unclear that this inequality in disability results from the disadvantage in socioeconomic status or military experiences that have been documented among AI/AN veterans. American Indian and Alaska Native veterans are also more likely to delay care due to difficulty in accessing care (Johnson et al., 2010). Additionally, AI/AN male veterans seem to have lower levels of income and more likely to be unemployed than white veterans (Department of Veterans Affairs, 2012; 2017; Holiday et al., 2006). In terms of education, AI/AN veterans have similar odds of holding bachelor’s degrees as other veterans but less likely to have advanced degrees (Department of Veterans Affairs, 2012). Yet, recent research demonstrates that there are no statistically significant differences between urban and rural AI/AN veteran health outcomes (Kaufman et al., 2020). Relative to white veterans, AI/AN veterans reported poorer physical and mental health outcomes, and urban AI/AN veterans were 72% more likely to report financial barriers to care compared to their urban white veteran peers (Kaufman et al., 2020; Westermeyer & Canive, 2013). While past research has indicated worse health of AI/AN veterans, the extent to which these disparities result from specific factors, especially experiences within the military, remains less clear. The military is a unique occupation with unique benefits (e.g., occupational training and educational benefits) and risks to subsequent health (e.g., exposure to combat and exposure to toxins); thus, accounting for military-related factors and experiences is imperative to understanding health disparities among veterans. Here, we contribute to the increasing body of research that documents disparities in health among veterans by analyzing the importance of socioeconomic status, health behaviors, and critical experiences that occur during military service (e.g., period of service and exposure to toxins or combat experience during military service).

Despite efforts by the federal government to address historical disadvantages (Fine-Dare, 2002), AI/ANs in general have the worst health and socioeconomic outcomes of almost any racial group in the United States (Indian Health Service, 2019). Although the data in this analysis cannot explicitly speak to the multitude of reasons these health inequities persist, research suggests a multitude of reasons, which include institutional discrimination acting through the global system of white supremacy (Allen, 2001) and which can be seen taking the form of government-endorsed policies of genocide and ethnocide, as well as the creation of and dishonoring of “treaties” by the US government (Dunbar-Ortiz, 2014). Other research suggests the present day subtle and overt racism against AI/AN peoples (Robertson, 2015), as well as the accumulation and effects of stress on health stemming from environmental factors such as perceived and real discrimination from being a minority (Thoits, 2010), contributes to health inequities in this population.

In light of the disproportionate rates of high military participation and of high rates of chronic illness, we are interested in exploring veteran health through examining the self-rated health and ADL limitations of AI/AN veterans compared to white veterans in a nationally representative sample of veterans. As AI/AN veterans differ systematically from white veterans in their socioeconomic status (Department of Veterans Affairs, 2012; 2017; Holiday et al., 2006; Montgomery et al., 2020), health behaviors, as well as access to health care (Kramer et al., 2009; Wallace et al., 2006), and experiences during military service (Beals et al., 2005), we analyze how statistically accounting for these differences influences any observed inequality in self-rated health and disability between AI/AN veterans and white veterans. We also analyze if the health disparities are concentrated in older adulthood. Thus, we not only contribute to past research on racial inequality among military veterans (Sheehan et al., 2015; Swed et al., 2018) and research on the health of the AI/AN veteran population (Kaufman et al., 2013; 2020; Koo et al., 2015) but also systematically analyze why such striking health disparities may be apparent and when they might occur.

## Methods

### Data

The data for this analysis came from the 2010 National Survey of Veterans (henceforth NSV). The NSV was commissioned by the Department of VA to plan for future programs that meet the needs of veterans (Department of Veterans Affairs, 2010). The NSV was a nationally representative, self-administered, mail-out-then-mail-back survey sent to six groups: active duty service members, active duty spouses, demobilized National Guard and Reserve members, veteran spouses, surviving spouses, and veterans. For the context of this analysis, we analyzed only veterans. We used the NSV due to the fact that it is nationally representative, had a large sample of veterans, and, most importantly, contains detailed information regarding the military service, socioeconomic attainment, and health of veterans. For our analysis, we excluded all those who reported a race other than white or AI/AN and those who were missing information on the health variables. Our final analytical sample was 7596. Unfortunately, we were unable to disaggregate AI/AN respondents by tribal affiliation.

### Variables and Regression Models

As we were interested in health differences between AI/ANs and whites (AI/AN persons were coded as “1” and non-Hispanic whites as “0”), we used two separate measures of health: self-rated health and ADLs. Self-rated health has been widely documented to be a reliable overall measure of health due to its high concordance with physicians’ reports and because it is a highly reliable predictor of mortality (Idler & Benyamini, 1997). Respondents were asked, “In general would you say your health is: *excellent*, *very good*, *good*, *fair*, or *poor*.” Consistent with past research regarding the health of veterans (e.g., Sheehan et al., 2015 and nonveterans, e.g., Luckhaupt et al., 2017), we dichotomized self-rated health so that *fair* and *poor* responses were coded “1” and *excellent*, *very good*, and *good* responses were coded “0.” We conducted additional analyses with “poor” health versus the other categories and found consistent inequity between AI/AN and white veterans. However, due to small cell sizes and unstable estimates, we do not report the findings. We dichotomized self-rated health rather than using the ordinal variable as the measure failed the parallel lines test (Hoffmann, 2004). We used logistic regression to model self-rated health.

We also used ADLs to measure health. Activities of daily living measure the ability to live and function independently. They describe collective fundamental skills that are required to independently care for oneself (Katz, 1983). Ultimately, when one is no longer able to do these tasks, they are no longer able to care for themselves, and, for veterans, they may qualify for Veterans Affairs’ Aid and Attendance benefit (Department of Veterans Affairs, 2020). The ADL measure asked respondents if during the past week they needed assistance to do the following activities: bathing, eating, getting out of bed or a chair, using the toilet, or walking. If the respondent indicated needing help on any of the activities, they were coded as “1”; if they did not need any assistance with any of the activities, they were coded “0.” We also used logistic regression to model ADLs.

In our analysis, we included basic demographic information and socioeconomic variables as controls. Specifically, we have years of age at survey coded continuously. Additionally, we included the gender of the veteran, with male coded “1” and female coded “0.” To maximize the sample size among AI/AN veterans, we included female veterans. As socioeconomic status is one way that AI/ANs are disadvantaged compared to whites, we included two measures. First, we included total household income as the following categorical variable: (1) $0–$34,999 as the reference category, (2) $35,000–$74,999, and (3) $75,000+. Similarly, we coded education as a categorical variable with (1) less than high school as the reference category, (2) high school, (3) some college, (4) college, and (5) professional degree. Similarly, we included marital status as a categorical variable with (1) married as the reference, (2) divorced, (3) widowed, and (4) never married.

Perhaps the greatest strength of the NSV compared to other large surveys in analyzing veterans is the detailed information collected regarding military service and individual health behaviors. We coded these questions in the following manner and consistent with previous research (Swed et al., 2018). First, we coded the age of entry as the following categorical variable: (1) 20 years and below as the reference, (2) 21–24 years, and (3) 25 years and older. We coded branch of service in a similar manner: (1) Army as the reference, (2) Navy, (3) Marines, (4) Air Force, and (5) other or multiple branches. We also included the duration of service coded with (1) 0–4 years served as the reference, (2) 5–19 years served, and (3) 20+ years served. We also included two combat covariates. First, we coded if they were ever in an active combat zone with “1” and “0” if they were not. The second combat variable was if they were ever exposed to dead or wounded people, which we coded as “1” if they were and “0” if not. We also included a variable which measured if the respondent was ever exposed to an environmental hazard, which we coded (1) they indicated definitely or probably being exposed as the reference, (2) probably not exposed, (3) definitely not exposed, and (4) unsure. Finally, we coded the most recent period of service as a categorical variable: (1) World War II through right before Vietnam as the reference, (2) Vietnam, and (3) any period after Vietnam. While the NSV has many strengths for analyzing veterans, it does lack a detailed collection of health behavior variables. However, it did contain information regarding the smoking status of the respondent, which we coded (0) never smoker as the reference, (1) past smoker, (2) current someday smoker, and (3) current everyday smoker.

We used Stata statistical software to conduct descriptive and multivariable analyses (Stata Statistical Software, 2015). We calculated descriptive statistics and confidence intervals for each of our measures. Next, we fit a series of logistic regression models predicting the dichotomized coded self-reported health. For ease of interpretability, we present our results in odds ratios (OR). In the first model, we analyzed AI/AN differences in self-rated health compared to whites, controlling for age and gender. In the second model, we additionally included educational attainment, household income, and marital status. In the third model, we additionally controlled for smoking, access to health care, and period of service. In the final model, we also added all the military service information: branch of service, age of entry, duration of service, combat experiences, and environmental exposures. We then implemented the same modeling procedure for one or more ADLs compared to 0 ADLs. Finally, as an additional analysis, we fit additional models predicting self-rated health and 1+ ADL with an interaction term between AI/AN and age specified in multiple ways. This allowed us to formally test if the health disparities are concentrated in certain age-groups. All of the analyses are weighted to account for the NSV sampling design. Missing data were accounted for with Stata’s multiple imputation suite (Allison, 2002).

## Empirical Results

### Descriptive Statistics

Descriptive statistics are presented in Table 1. Our total sample size was 7596 where 208 were AI/AN and 7388 were white. With our reported health measures, 33% of AI/ANs reported fair/poor self-rated health, and 14% reported having one or more ADLs. Among whites, 26% reported fair/poor self-rated health, and 8% reported having one or more ADLs. In terms of poor health, 14% of AI/AN veterans reported poor health versus 8% of white veterans. The average age of the AI/AN veterans was 56.52, and 62.33 was the average age of the white veterans. The median age for Native veterans was 61 and 65 for white veterans. Over 90% of our sample was male for both racial groups. Thirty-eight percent of our sample reports a household income of $35,000 to $74,999, followed by the reference category of $0 to $34,999, and 28% of our sample reported having a household income greater than $75,000. In terms of education among our sample, 29% had some college followed by holding a college degree, high school degree (26%), professional degree (12%), and least common was less than high school (6%). Sixty-one percent of AI/ANs were married, and 73% of whites were married. Among AI/ANs, the most common smoking status was never smoking; for whites, it was being a past smoker. In terms of VA healthcare service use, 61% of AI/ANs never used the VA healthcare services, 15% have used it but not in the past 6 months, and 24% have used the VA healthcare services in the past 6 months. For white veterans, 73% have never used the VA healthcare services, followed by 18% who used the services within the last 6 months, and 9% who have used the services in the past but not within the last 6 months. Overall, AI/AN veterans tend to be younger, have lower income, and more likely to report poorer health than white veterans.

**Table 1.** Descriptive Statistics, of Health, Demographic, Socioeconomic Factors, Military, and Health.

	NSV Veterans			American Indian/Alaska Native Peoples			Non-Hispanic White		
	(*N* = 7596)			(*N* = 208)			(*N* = 7388)		
	Mean or Proportion	95% CI		Mean or Proportion	95% CI		Mean or Proportion	95% CI	
Reported health									
Fair/poor	0.26	0.25	0.27	0.33	0.26	0.41	0.26	0.25	0.27
Poor	0.07	0.07	0.08	0.15	0.09	0.20	0.07	0.06	0.08
Limitation in one or more activities of daily living	0.08	0.07	0.09	0.14	0.08	0.20	0.08	0.07	0.08
Male	0.93	0.92	0.94	0.91	0.85	0.96	0.93	0.92	0.94
Age	62.16	61.69	62.62	56.52	53.94	59.09	62.33	61.85	62.80
Household income									
$0–$34,999 (ref)	0.33	0.32	0.34	0.48	0.40	0.56	0.33	0.32	0.34
$35,000–$74,999	0.38	0.37	0.40	0.26	0.20	0.32	0.39	0.37	0.40
$75,000+	0.28	0.27	0.30	0.26	0.19	0.33	0.29	0.27	0.30
Educational attainment									
Less than high school degree (ref)	0.06	0.05	0.06	0.06	0.02	0.09	0.06	0.05	0.06
High school degree	0.26	0.25	0.27	0.21	0.14	0.27	0.26	0.25	0.27
Some college	0.29	0.28	0.30	0.38	0.30	0.46	0.29	0.28	0.30
College degree	0.27	0.26	0.28	0.25	0.18	0.31	0.27	0.26	0.28
Professional degree	0.12	0.11	0.13	0.10	0.06	0.15	0.12	0.12	0.13
Marital status									
Married (ref)	0.73	0.72	0.74	0.61	0.53	0.69	0.73	0.72	0.74
Divorced	0.14	0.13	0.15	0.23	0.16	0.29	0.13	0.12	0.14
Widowed	0.07	0.06	0.07	0.04	0.01	0.06	0.07	0.06	0.07
Never married	0.07	0.06	0.08	0.13	0.06	0.20	0.07	0.06	0.08
Smoking									
Never smoker (ref)	0.35	0.34	0.36	0.36	0.28	0.43	0.35	0.34	0.36
Past smoker	0.47	0.46	0.48	0.33	0.25	0.40	0.48	0.46	0.49
Current someday smoker	0.05	0.04	0.05	0.04	0.01	0.07	0.05	0.04	0.05
Current everyday smoker	0.13	0.12	0.14	0.28	0.20	0.35	0.13	0.12	0.14
Veteran Administration health care									
Never used (ref)	0.73	0.72	0.74	0.61	0.54	0.69	0.73	0.72	0.74
Used, but not in the past 6 months	0.09	0.08	0.10	0.15	0.09	0.21	0.09	0.08	0.10
Used in the past 6 months	0.18	0.17	0.19	0.24	0.16	0.31	0.18	0.17	0.19
Most recent period of service									
WWII-Vietnam (ref)	0.33	0.32	0.34	0.19	0.13	0.24	0.34	0.32	0.35
Vietnam	0.29	0.28	0.30	0.27	0.21	0.34	0.29	0.28	0.30
Post-Vietnam	0.38	0.36	0.39	0.54	0.46	0.62	0.37	0.36	0.39
Branch of service									
Army (ref)	0.44	0.43	0.46	0.33	0.26	0.41	0.45	0.44	0.46
Navy	0.23	0.22	0.24	0.25	0.18	0.33	0.23	0.22	0.24
Marines	0.09	0.08	0.10	0.15	0.09	0.22	0.09	0.08	0.10
Air Force	0.20	0.19	0.21	0.20	0.14	0.26	0.20	0.19	0.21
Other/multiple branches	0.04	0.03	0.04	0.06	0.03	0.09	0.04	0.03	0.04
Age of entry									
20 and below (ref)	0.63	0.62	0.64	0.71	0.63	0.78	0.63	0.62	0.64
21–24	0.31	0.29	0.32	0.26	0.19	0.33	0.31	0.30	0.32
25+	0.06	0.06	0.07	0.04	0.00	0.07	0.06	0.06	0.07
Duration of service									
0–4 (ref)	0.74	0.72	0.75	0.67	0.60	0.75	0.74	0.73	0.75
5–19 years	0.19	0.18	0.20	0.22	0.15	0.29	0.19	0.17	0.20
20+ years	0.08	0.07	0.08	0.11	0.07	0.15	0.08	0.07	0.08
Combat experiences									
Served in combat/war zone	0.34	0.32	0.35	0.40	0.32	0.47	0.33	0.32	0.35
Exposed to death/trauma	0.34	0.32	0.35	0.45	0.37	0.53	0.33	0.32	0.34
Environmental hazards									
Definitely/probably exposed (ref)	0.23	0.22	0.24	0.39	0.32	0.47	0.22	0.21	0.23
Probably not exposed	0.27	0.26	0.28	0.23	0.17	0.30	0.27	0.26	0.29
Definitely not exposed	0.30	0.29	0.32	0.14	0.09	0.20	0.31	0.30	0.32
Unsure	0.20	0.18	0.21	0.23	0.16	0.30	0.19	0.18	0.20

Source: *National Survey of Veterans 2010.*

*Note.* Missing data were handled by Stata’s multiple imputation suite.

In terms of period of service, our sample of AI/AN veterans served post-Vietnam (54%), Vietnam era (27%), and WWII-Vietnam era (19%), and for our sample of white veterans, 37% served in the post-Vietnam era, followed by WWII-Vietnam era (34%), and then Vietnam era (29%). In our sample, Army was the most common branch to have served followed by the Navy, Air Force, and Marines. The majority of our sample had a military age of entry 20 years and below. Sixty-seven percent of the AI/AN sample served for 0–4 years, compared to 74% of whites. Approximately 40–45% of AI/AN veterans had combat experiences, whether that be served in combat/war zone or were exposed to death/trauma, versus approximately 33% of whites. Veterans who reported definitely/probably exposed to environmental hazards accounted for 39% of the AI/AN sample and 22% of the white sample.

### Logistic Regression Results for Self-Rated Health

Table 2 presents our logistic regression findings that predict “Fair” or “Poor” self-rated health among veterans. Controlling for gender and age, we found that AI/AN veterans are 73% (i.e., OR = 1.73, 95% CI: [1.22, 2.44]) more likely to report fair/poor health than white veterans (see Model 1). In Model 2, we introduced socioeconomic controls and found that AI/AN veterans were 54% (i.e., OR = 1.54, 95% CI: [1.07, 2.20]) more likely to report fair/poor health relative to whites. We also found that increased levels of education attainment and household income were associated with lower likelihood of reporting fair/poor health. Model 3 additionally controlled for health behaviors, and we found that AI/AN veterans have 1.41 higher odds of reporting fair/poor health than whites; however, after accounting for access to smoking, access to health care, and period of service, we found that the differences between AI/AN veterans and white veterans were no longer statistically significant (OR = 1.41, 95% CI: [0.97–2.07]). Current everyday smokers were more likely to report fair/poor health than those who have never smoked. Veterans who have used VA healthcare services in the past 6 months were more likely to report fair/poor health relative to those who have never used VA health care. Finally, in this model, we found that Vietnam veterans were more likely to report fair/poor health relative to WWII-Vietnam veterans. In Model 4, we included additional military service differences, and we saw the greatest reduction in the likelihood of AI/AN veterans to report fair/poor health relative to white veterans and the difference remains nonsignificant. In terms of combat experiences, veterans who served in a combat/war zone or were exposed to death were more likely to have reported fair/poor health relative to those veterans who have not had those combat experiences. Known exposure to environmental hazards also increased the odds of reporting fair/poor health.

**Table 2.** Odds Ratios From Logistic Regression Models Predicting “Fair” or “Poor” Self-Rated Health Among Veterans.

	Model 1				Model 2				Model 3				Model 4			
	OR	95% CI			OR	95% CI			OR	95% CI			OR	95% CI		
Race/ethnicity																
White (ref)	—				—				—				—			
American Indian/Alaska Native peoples	1.73	1.22	2.44	**	1.54	1.07	2.20	*	1.41	0.97	2.07		1.28	0.88	1.88	
Gender																
Female (ref)	—				—				—				—			
Male	1.27	0.96	1.69		1.32	0.99	1.77		1.21	0.90	1.64		1.07	0.78	1.46	
Age	1.03	1.02	1.03	***	1.02	1.02	1.03	***	1.04	1.03	1.04	***	1.04	1.03	1.05	***
Household income																
$0–$34,999 (ref)					—				—				—			
$35,000–$74,999					0.60	0.52	0.70	***	0.71	0.60	0.82	***	0.69	0.59	0.81	***
$75,000+					0.31	0.26	0.38	***	0.41	0.33	1.04	***	0.40	0.33	0.50	***
Educational attainment																
Less than high school degree (ref)					—				—				—			
High school degree					0.61	0.49	0.77	***	0.62	0.49	0.78	***	0.64	0.50	0.81	***
Some college					0.47	0.37	0.59	***	0.47	0.36	0.59	***	0.46	0.36	0.59	***
College degree					0.36	0.28	0.46	***	0.36	0.28	0.47	***	0.37	0.29	0.48	***
Professional degree					1.06	0.19	0.35	***	0.28	0.21	0.38	***	0.29	0.21	0.40	***
Marital status																
Married (ref)					—				—				—			
Divorced					1.20	1.00	1.44		1.02	0.84	1.23		1.00	0.82	1.21	
Widowed					0.84	0.68	1.05		0.87	0.69	1.09		0.85	0.67	1.07	
Never married					1.06	0.80	1.42		1.05	0.78	1.42		1.12	0.83	1.52	
Smoking																
Never smoker (ref)									—				—			
Past smoker									1.29	1.12	1.50	**	1.22	1.06	1.42	**
Current someday smoker									1.95	1.41	2.68	***	1.79	1.30	2.46	***
Current everyday smoker									2.16	1.76	2.65	***	2.08	1.69	2.56	***
Veteran Administration health care																
Never used (ref)									—				—			
Used, but not in the past 6 months									1.65	1.35	2.02	***	1.50	1.22	1.85	***
Used in the past 6 months									2.67	2.28	3.13	***	2.35	2.00	2.77	***
Most recent period of service																
WWII-Vietnam (ref)									—				—			
Vietnam									1.62	1.35	1.94	***	1.29	1.05	1.58	*
Post-Vietnam									1.43	1.07	1.90	*	1.10	0.77	1.58	
Branch of service																
Army (ref)													—			
Navy													0.76	0.64	0.89	**
Marines													0.93	0.72	1.20	
Air Force													0.89	0.75	1.05	
Other/multiple branches													0.90	0.65	1.25	
Age of entry																
20 and below (ref)													—			
21–24													0.74	0.64	0.86	***
25+													1.12	0.82	0.82	
Duration of service																
0–4 (ref)													—			
5–19 years													1.13	0.93	1.36	
20+ years													1.12	0.83	1.51	
Combat experiences																
Served in combat/war zone													0.79	0.67	0.94	**
Exposed to death													1.43	1.21	1.69	***
Environmental hazards																
Definitely/probably exposed (ref)													—			
Probably not exposed													0.59	0.49	0.71	***
Definitely not exposed													0.47	0.38	0.57	***
Unsure													0.71	0.59	0.86	***
Constant	0.05	0.03	0.07	***	0.23	0.14	0.38	***	0.04	0.02	0.09	***	0.09	0.04	0.22	***

Source: *National Survey of Veterans 2010.*

* *p* < 0.05; ** *p* < 0.01; *** *p* < 0.001.

*Note.* Missing data were handled by Stata’s multiple imputation suite.

### Logistic Regression Results for ADLs

Table 3 shows a set of progressive logistic regression models to predict one or more ADLs among our veteran sample. In our first model, we found that AI/AN veterans have 2.31 higher odds (95% CI: [1.35, 3.94]) of experiencing one or more ADLs relative to white veterans, controlling for gender and age. In Model 2, we added socioeconomic control variables, and we saw a slight reduction to 2.08 higher odds (95% CI: [1.22, 3.56]). For socioeconomic variables, we saw an expected trend of higher levels of income and education having lower odds of experiencing an ADL relative to those with lower levels of income and education. Among our health behavior variables, veterans who are current someday smokers were more likely to report an ADL than veterans who have never smoked. Veterans who report using VA healthcare services within the past 6 months were more likely to report an ADL than veterans who have never used the VA healthcare services. In Model 4, the disparity in ADLs between AI/AN and white veterans reduced from 2.31 in Model 1 to 1.81 when controlling for military service factors. However, unlike self-reported health, the differences remained statistically significant (OR = 1.81, 95% CI: [1.06–3.10]). We saw many of the same trends across our variables. We did not see statistical significance among the veterans in terms of branch of service, age of entry, or duration of service variables. However, we did see that veterans who have combat experiences that involve exposure to death have 1.65 increased odds (95% CI: [1.26, 2.15]) of reporting an ADL than veterans who had not had this type of combat experience. Exposure to environmental hazards also increased likelihood of an ADL, where those who did not have a known exposure have lower odds of reporting an ADL than veterans who definitely/probably were exposed to an environmental hazard.

**Table 3.** Odds Ratios From Logistic Regression Models Predicting 1+ Activity of Daily Living Limitation Among Veterans.

	Model 1				Model 2				Model 3				Model 4			
	OR	95% CI			OR	95% CI			OR	95% CI			OR	95% CI		
Race/ethnicity																
White (ref)	—				—				—				—			
American Indian/Alaska Native peoples	2.31	1.35	3.94	**	2.08	1.22	3.56	**	1.98	1.15	3.40	*	1.81	1.06	3.10	*
Gender																
Female (ref)	—				—				—				—			
Male	0.68	0.45	1.00		0.69	0.46	1.03		0.73	0.48	1.11		0.63	0.41	0.96	
Age	1.03	1.02	1.04	***	1.02	1.01	1.03	**	1.02	1.01	1.04	**	1.02	1.00	1.04	*
Household income																
$0–$34,999 (ref)					—				—				—			
$35,000–$74,999					0.55	0.43	0.70	***	0.65	0.51	0.83	**	0.64	0.50	0.82	***
$75,000+					0.27	0.18	0.40	***	0.36	0.24	0.54	***	0.36	0.24	0.54	***
Educational attainment																
Less than high school degree (ref)					—				—				—			
High school degree					0.62	0.45	0.87	**	0.65	0.47	0.91	*	0.68	0.48	0.97	*
Some college					0.58	0.42	0.81	**	0.60	0.43	0.84	**	0.59	0.42	0.84	**
College degree					0.53	0.37	0.74	***	0.54	0.38	0.77	**	0.54	0.37	0.79	**
Professional degree					0.36	0.23	0.56	***	0.37	0.23	0.60	***	0.36	0.22	0.59	***
Marital status																
Married (ref)					—				—				—			
Divorced					0.84	0.61	1.16		0.79	0.57	1.08		0.78	0.56	1.08	
Widowed					0.87	0.63	1.21		0.83	0.60	1.16		0.80	0.58	1.11	
Never married					0.81	0.46	1.41		0.74	0.42	1.30		0.75	0.42	1.34	
Smoking																
Never smoker (ref)									—				—			
Past smoker									0.99	0.78	1.25		0.95	0.75	1.20	
Current someday smoker									1.90	1.22	2.98	**	1.79	1.14	2.82	*
Current everyday smoker									1.18	0.82	1.70		1.17	0.81	1.68	
Veteran Administration health care																
Never used (ref)									—				—			
Used, but not in the past 6 months									1.73	0.00	1.25	**	1.55	1.11	2.15	**
Used in the past 6 months									2.46	1.96	3.08	***	2.10	1.65	2.66	***
Most recent period of service																
WWII-Vietnam (ref)									—				—			
Vietnam									0.81	0.58	1.12		0.54	0.38	0.77	**
Post-Vietnam									1.19	0.73	1.93		0.65	0.37	1.17	
Branch of service																
Army (ref)													—			
Navy													0.91	0.69	1.18	
Marines													0.88	0.57	1.37	
Air Force													1.00	0.74	1.36	
Other/multiple branches													1.17	0.74	1.85	
Age of entry																
20 and below (ref)													—			
21–24													0.92	0.72	1.19	
25+													1.39	0.91	2.11	
Duration of service																
0–4 (ref)													—			
5–19 years													1.15	0.84	1.57	
20+ years													1.41	0.91	2.18	
Combat experiences																
Served in combat/war zone													0.90	0.67	1.20	
Exposed to death													1.65	1.26	2.15	***
Environmental hazards																
Definitely/probably exposed (ref)													—			
Probably not exposed													0.59	0.43	0.80	**
Definitely not exposed													0.53	0.39	0.74	***
Unsure													0.78	0.57	1.05	
Constant	0.02	0.01	0.04	***	0.11	0.05	0.25	***	0.04	0.01	0.18	***	0.14	0.03	1.05	*

Source: *National Survey of Veterans 2010.*

* *p* < 0.05; ** *p* < 0.01; *** *p* < 0.001.

*Note.* Missing data were handled by Stata’s multiple imputation suite.

### Sensitivity Analyses to Test Age Differences

As an additional sensitivity analysis (results available upon request), we analyzed if there were significant differences in risk of reporting “fair” or “poor” health or 1+ ADL across age and, specifically, in older adulthood. To investigate this issue, we fit three interaction models for each outcome only controlling for gender. For the first, we interacted AI/AN with age (specified continuously) and found that the interaction was not significant for self-rated health or ADLs. For the second model, we created a dummy variable with “1” coded as those who were 50 and older and “0” if otherwise. Again, the interaction term was not statistically significant suggesting that the health disparities were not pronounced among older adults. Finally, we coded an additional dummy variable with those aged 65+ coded “1” and those 64 and younger coded “0.” Once again, the interaction model was not statistically significant, suggesting there were little differences in the extent of the health disparities across age or older adulthood.

## Discussion

American Indian and Alaska Native peoples have a long history of participating in the US military and continue to be present today. Indeed, a disproportionately high percentage of veterans, relative to their nonveteran population, identify as AI/AN (Department of Veterans Affairs, 2012, 2017). Extant literature suggests that AI/AN veterans carry higher burdens of disease, including more likely to report a disability and/or poorer mental health outcomes than white veterans (Department of Veterans Affairs, 2012; 2017; Koo et al., 2015). Our study contributes to this past research by examining self-rated health and ADLs of AI/AN veterans relative to white veterans while systematically analyzing how the health disparities are influenced by different groups of controls. Specifically, we contribute to past research by being able to consider age of entry, branch of service, duration of service, combat experience, exposure to environmental hazards, period of service, and health behaviors. In terms of self-reported health, we found that there were differences between AI/AN veterans and white veterans, but these differences were statistically significant only when smoking, access to care, and period of service were controlled for. There is rich literature that has documented AI/AN veterans’ inability to access quality VA healthcare services, and our findings suggest this influences their self-reported health (Johnson et al., 2010).

The inclusion of ADLs allows us to examine an aspect of quality of life for AI/AN veterans—the ability to independently care for oneself. As with the majority of literature, ADLs are often focused on elderly populations (Katz, 1983); however, Sheehan et al.’s (2015) study on racial and ethnic minority veterans suggest that it is a useful measure to understand variation in health status among veterans. Yet, ADLs are especially important for AI/AN persons who report having functional limitations younger than other racial and ethnic groups (Altman & Rasch, 2003; Fuller-Thomson & Minkler, 2005; Moss et al., 2004), and we argue that analyzing ADLs along with self-reported health adds a crucial dimension to understanding the health status and life quality of AI/AN veterans.

In terms of the general description of the study sample, AI/AN veterans have a higher prevalence of reported fair/poor health, reporting one or more ADLs, and are younger than white veterans. AI/AN veterans have lower levels of income with 48% of the sample having a household income of $0 to $34,999 compared to 33% of white veterans. For our health behavior measures, AI/AN and white veterans have similar trends of current and past smoking behavior and similar use of the VA healthcare services (for further description of AI/AN veteran healthcare service use, see Tsai et al., 2014). American Indian and Alaska Native veterans are slightly more likely to have combat experiences relative to white veterans, but the difference is not statistically significant. In terms of environmental hazards, AI/AN veterans have a higher prevalence of being exposed to environmental hazards than white veterans. Taken all together, AI/AN veterans are at higher risk of poor health outcomes due to more exposure to combat and environmental dangers and lower socioeconomic status despite being younger than white veterans and having similar health behaviors to white veterans.

Our analysis uncovered significant differences between whites and AI/ANs in self-rated health in our early models, which is supported by previous literature that has also found that AI/AN men are more likely to report fair/poor health relative to white men (Denny et al., 2005). In our accounting of smoking status, access to health care, and period of service, we found that the differences between AI/AN veterans and white veterans were no longer statistically significant. These findings suggest a mediating role of these control variables. Telehealth has the potential of increasing healthcare access for AI/AN populations alongside investment of technological infrastructure in tribal communities to increase regular access to telephone and internet (Kruse et al., 2016). Finally, when controlling for branch of service, age of entry, and exposure to combat experiences and environmental hazards during service period, we found the greatest reduction in the likelihood of AI/AN veterans to report fair/poor health relative to white veterans. Previous literature has also found a strong association between combat exposure and self-reported health among veterans (Schnurr & Spiro, 1999). To undertake health inequities among veterans, future research should consider the influence of multiple mechanisms and life experiences that impact AI/AN health.

Our results for ADL has revealed an important health indicator, where across all our logistic regression models, AI/AN veterans have approximately 2 times the odds of experiencing an ADL relative to white veterans. It further bolsters the idea that racial/ethnic minority veterans are in poorer health than their white veteran counterparts (Sheehan & Hayward, 2019). This finding stands in spite of the fact that the health of all veterans is relatively comparable across race/ethnic groups as individuals enter the military and minorities generally smoke less than whites (Teachman, 2011; Chisick et al., 1998). The implications of this finding are stark as recent research has documented that veterans with a disability are at significantly higher risk of mortality (Landes et al., 2019). Future research should further explore the age of onset of ADLs among racial and ethnic minority veterans and the military and social factors that may increase their risk of experiencing an ADL. The importance of understanding ADL onset and occurrence only elucidates the quality of life of veterans. Furthermore, additional resources are needed from the Veteran Administration or family to provide caregiving to veterans and especially to AI/AN veterans. Kramer et al. (2015) have found that collaborations between the Veteran Health Administration and Indian Health Service improve access to noninstitutional long-term care for Native veterans. It is briefly worth noting that we did not find any age differences; however, it is unclear if this is due to our sample size. Future research with larger samples should consider further investigating age and cohort differences in health disparities.

There are some important limitations to our study. Most importantly, the dataset we used was cross-sectional, giving us only a snapshot of an incredibly complex life course process of health status for veterans (MacLean & Elder, 2007). However, we are unaware of any longitudinal surveys of health that have sizable numbers of AI/AN veterans that are generalizable to the entire veteran population and contain such detailed military covariates. Second, we are also missing important variables that have been associated with veteran health. For example, the NSV did not include data on highest achieved rank. Officers have better health and live longer than their lower ranking non-officer peers (Edwards, 2008; MacLean & Edwards, 2010). We attempted to minimize this by including time served, educational attainment, and age of enlistment. Next, we did not have any variables regarding alcohol consumption. Some have asserted that veterans have worse health than nonveterans due to their higher levels of alcohol consumption. Despite not having comprehensive health behavior measures, our study continues to illuminate the role of military experiences and specific social factors in the health status and health outcomes of veterans.

American Indian and Alaska Native peoples have a long history of honorably serving in the US military yet experience some of the highest health disparities. This analysis demonstrates significant differences between whites and AI/ANs self-rated health and ADLs. Our findings correspond with past research that increasingly documents that the military is not immune from racial/ethnic inequities in health. Even when controlling for such variables like age and socioeconomic status, AI/AN veterans are still more likely to have poorer self-rated health, and AI/ANs have approximately 2 times the odds of reporting an ADL limitation or impairment relative to white veterans, net of controls for age, health behaviors, socioeconomic status, and military factors. The literature demonstrates that AI/AN veterans are being institutionally underserved and do not have the same access to health care as their white counterparts. Although future research is needed on AI/AN veteran outcomes, including more that focuses on their legacy of resiliency which our data could not speak to, one thing is abundantly clear: AI/AN veterans’ health outcomes must be addressed as a piece of honoring their sacrifice to the United States and its military.
